# Mental health support for children and adolescents with hearing loss: scoping review

**DOI:** 10.1192/bjo.2021.1045

**Published:** 2021-12-06

**Authors:** Nathaniel Scherer, Tess Bright, David John Musendo, Timothy O'Fallon, Chris Kubwimana, Julian Eaton, Ritsuko Kakuma, Tracey Smythe, Sarah Polack

**Affiliations:** International Centre for Evidence in Disability, London School of Hygiene & Tropical Medicine, UK; DeafReach, UK; Centre for Global Mental Health, London School of Hygiene & Tropical Medicine, UK; International Centre for Evidence in Disability, London School of Hygiene & Tropical Medicine, UK

**Keywords:** Childhood experience, outcome studies, psychosocial interventions, rehabilitation, primary care

## Abstract

**Background:**

Children with hearing loss are at increased risk of mental health conditions, including behavioural problems, but there is limited evidence about available mental health support.

**Aims:**

We aimed to map the evidence on mental health support for children and adolescents with hearing loss.

**Method:**

Medline, Embase, PsycINFO and grey literature databases were searched until April 2021. Articles of any study design were eligible if they described an intervention supporting the mental health of children with hearing loss. No restrictions were placed on geography or publication date. Four reviewers independently screened results by title, abstract and full text. Study characteristics and outcome data were extracted, with results narratively synthesised.

**Results:**

From 5629 search results, 27 articles were included. A large majority of the studies (81%, *n* = 22) were from high-income settings, with two-thirds (67%, *n* = 18) conducted in the USA. Less than half (41%, *n* = 11) of the articles adopted experimental research designs, and the majority of studies included small samples. The interventions presented were diverse, with the majority either therapy based (30%, *n* = 8) or skills training (30%, *n* = 8). Interventions included ice-skating, parent–child interaction therapy and resilience training. When measured, interventions demonstrated at least some evidence of effectiveness, although this was not always assessed with gold-standard methodology.

**Conclusions:**

The evidence is lacking in breadth, study quality and geographical spread. That said, what is available indicates a range of effective approaches to support the mental health of children with hearing loss. Additional research is needed to improve the breadth of evidence on mental health support for this population.

## Background

As of 2019, an estimated 20% of the global population (or 1.57 billion) have hearing loss, with 430 million having moderate-to-complete hearing loss.^1^ This is a 79% increase in reported rates from 1990, and by 2050, an estimated 2.45 billion people are estimated to have hearing loss, with 698 million having moderate-to-complete hearing loss. Although hearing loss is more common among older adults, there are approximately 70 million children aged 0–15 with hearing loss across the world.^1^

Children with hearing loss may experience language deprivation, impacting development, communication and socioemotional skills.^2,3^ As a result, children with hearing loss are at increased risk of mental health conditions, such as anxiety and depression, with several studies demonstrating significantly higher prevalence of these conditions among children with hearing loss, compared with children without hearing loss.^4–9^ Evidence also shows that children with hearing loss are more likely to experience behavioural problems, including conduct and hyperactivity disorders.^10,11^ Half of mental health conditions start by the age of 14, yet these often go undetected and untreated.^12^ Among the general population, these conditions in childhood are associated with an increased risk of mental health concerns in adulthood, lower family income, lower probability of employment and lower probability of being married.^13,14^ Mental health conditions may also further disrupt ongoing child development, a challenge already present for children with hearing loss. Addressing mental health concerns, while promoting emotional, behavioural and psychological well-being, is imperative among children with hearing loss, in order to support a healthy childhood and reduce the risk of adverse experiences in adulthood.

Youth mental health has received growing attention in recent years, as evidenced by the inclusion of 104 network meta-analyses and meta-analyses of randomised controlled trials (RCTs) on mental health interventions for children and adolescents in a recent umbrella review published in 2021.^15^ However, there is limited synthesised evidence on interventions and types of support available for children with hearing loss that promote psychological, emotional and behavioural well-being, prevent mental health conditions and treat conditions that do arise. In 2019, a systematic review on the assessment and treatment of behavioural disorders in children with hearing loss found limited evidence of interventions to address behavioural problems.^11^ Evidence on interventions available for other mental health conditions is lacking. Information on the types of interventions applied, their characteristics and evidence of their effectiveness is needed to inform support programmes for children with hearing loss, whether these be in school, the community or delivered through the healthcare system.

## Aims

This study aimed to systematically identify and map the evidence on mental health support for children and adolescents with hearing loss. We aimed to answer the following questions.

- What are the characteristics of the available literature describing mental health support for children with hearing loss?
- What mental health interventions and support have been provided or evaluated for children with hearing loss?
- What evidence is available on the effectiveness of these interventions?
- What are the current gaps in the available evidence?

## Method

With expected limited and heterogeneous data, we chose to conduct a scoping review, rather than an alternative evidence synthesis, in order to map the available evidence.^16^ The protocol for the review was registered on the Open Science Framework on 3 March 2020 (osf.io/8qdbz/). Ethics approval was obtained from the London School of Hygiene & Tropical Medicine Research Ethics Committee (Ref: 19144).

The scoping review has been conducted and reported according to the PRISMA Extension for Scoping Reviews (PRISMA-ScR) guideline and followed Arksey & O'Malley's framework for scoping reviews.^17,18^ We conducted the review across five stages: (a) identifying the research question; (b) identifying relevant studies; (c) study selection; (d) charting the data; and (e) collating and summarising the results.

### Eligibility criteria

- Population: children aged 6–18, with diagnosed, proxy-report or self-reported hearing loss, of any severity. Included participants did not need to have a diagnosis of a mental disorder.
- Intervention: any initiative designed to improve the mental health and well-being of children with hearing loss, including interventions developed specifically for children with hearing loss or those adapted from interventions aimed at the general population. Interventions promoting developmental skills and protective factors, such as emotional regulation, resilience and self-esteem, were included if connection was made to child mental health and well-being. Interventions were also included if they addressed behavioural problems or disorders, such as hyperactivity or aggression. Activities could be focused on promotion, prevention or treatment, such as social interventions, skills development, targeted recreational activities and therapies. They could be delivered by any personnel in any setting. Interventions for parents, caregivers or other adults (for example teachers) were included if children with hearing loss were an intended beneficiary. Similarly, interventions were included if provided to a diverse group, as long as children with hearing loss were one of the beneficiaries.
- Comparator: studies with and without a control or comparison group were included. If a control or comparator is present, they must also be children with hearing loss of the same age, but who have not received the intervention.
- Outcomes: studies with or without assessment of any outcome were included. Where applicable, outcomes of interest included scores on mental health screening tools, acceptability/feasibility, cost-effectiveness and other reasonable data.
- Study design: published literature of any study design (quantitative, qualitative and mixed methods). Descriptive literature (i.e. without research methods applied) was included, if sufficient detail was provided on the intervention(s) available for children with hearing loss. Reviews and opinion pieces were excluded. There were no restrictions placed on geographic location, although articles needed to be in English. There were no limits placed on publication date. Grey literature, including dissertations and conference presentations, was included. Only articles with an available full text were included.

### Search strategy

Articles were identified through a systematic search of Medline, Embase and PsycINFO. The search was initially conducted on 27 April 2020 and updated on 27 April 2021. An example of the search strategy is available in Supplementary File 1 available at https://doi.org/10.1192/bjo.2021.1045. Reference lists of each included study were examined in search of additional articles for inclusion.

The search for grey literature was conducted through OpenGrey and Google Scholar. Additionally, experts in this field were contacted for recommendations of known reports, and the websites of notable disability and hearing loss organisations were manually searched (Supplementary File 2).

### Study selection

Authors N.S., T.B., D.J.M. and T.O.’F. independently screened all titles and abstracts against the eligibility criteria. Each record was screened by two reviewers. Eligible full-text articles were then independently reviewed by two reviewers. Although excluded from the final synthesis, systematic reviews and full-length books identified in the database search were manually screened by full-text, with included articles and book chapters selected for full-text screen, if relevant. Records identified from the reference lists of included articles were also filtered into the full-text screening. Discrepancies at any stage were discussed between the two reviewers, with a third and fourth reviewer consulted if needed. This review process was conducted using Covidence software.

### Data extraction and charting

N.S. extracted the data for each study, using a custom form, developed in Excel. This extraction form was first piloted on three included articles, with amendments made as necessary. T.B., D.J.M. and T.O.’F. each independently reviewed one-third of data extracted. Discrepancies were discussed and resolved with N.S., with support from a third reviewer if needed. Data extracted included:

- Publication details: author, year of publication, title, country, aims/objectives, study design.
- Characteristics of mental health support: type (promotion, prevention, treatment), intended outcome, setting, delivery agent, intervention components.
- Outcomes: type (effectiveness, feasibility, acceptability, service-delivery related), measurement tools, findings (both narrative and statistical).

### Quality assessment

For an assessment of risk of bias, we used the critical appraisal tools from the Joanna Briggs Institute (JBI). There are various tools provided by JBI, designed for use with different designs, such as case report or RCT. Each tool can be used to determine the extent to which a study has addressed the possibility of bias in its design, conduct and analysis, via a series of relevant questions and standardised responses.^19^

Studies were assessed by N.S., with scores reviewed by S.P. Based on the assessment against a JBI checklist, each study was rated as having high, medium or low risk of bias.

- Low risk: all or almost all of the criteria were fulfilled, and those that were not fulfilled were thought unlikely to alter the conclusions.
- Medium risk: some of the criteria were fulfilled, and those not fulfilled were thought unlikely to alter the conclusions of the study.
- High risk: few or no checklist criteria were fulfilled, and the conclusions of the study were thought likely to alter if these had been met.

### Synthesis of results

Under the umbrella of mental health, the focus of this review, we have grouped articles by interventions that address psychological well-being and behavioural problems. Psychological well-being, in this instance, refers broadly to emotional well-being and mental health, and includes diagnosis or symptoms of mental health conditions, as well as related domains, such as resilience and self-esteem. Behavioural problems include disorders and concerns relating to disruptive and challenging behaviours, such as aggression, impulsivity and defiance. Specific behavioural disorders include attention-deficit hyperactivity disorder or oppositional defiant disorder.

Under these two subheadings, we grouped articles by intervention type. Findings have been summarised narratively, with the focus on the characteristics of the interventions. Information on effectiveness has been narratively presented, where this information was available.

When data presented in an article is disaggregated by age, with participants older or younger than the inclusion criteria included, only that attributable to children within the age range of 6–18 years was included in the synthesis.

## Results

**Fig. 1:**
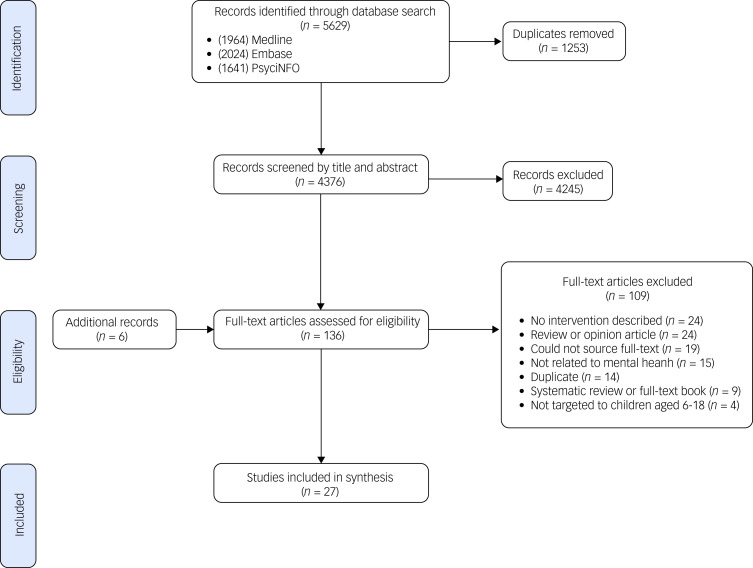
PRISMA flow chart.

The database search generated 5629 results, from which 1253 duplicates were removed. In total, 4376 records were screened by title and abstract, from which 4245 were excluded based on the criteria above (Fig. 1). There were 130 full-text articles that were eligible for screening, with 6 additional records included from systematic reviews screened at full text (*n* = 3), records identified via the reference lists of included articles (*n* = 2) and the grey literature (*n* = 1). From 136 full texts assessed, 27 articles were included in the synthesis.^20–46^

### Study characteristics

Of the 27 included articles, 18 (67%) come from North America, and specifically the USA (Table 1). Of the remaining, four (15%) were conducted in Europe, four (15%) the Middle-East, and one (4%) in Asia. None were from Africa, Latin America or Australasia. Nine (33%) were published after the year 2010, with 18 (67%) prior to 2010. Eleven (41%) were published prior to the year 2000.

**Table 1:** Summary of article characteristics

Variable and category	*n*	%
Region		
Asia	1	4
Europe	4	15
Middle-East	4	15
North America	18	67
Country		
Finland	1	4
India	1	4
Iran	3	11
Poland	1	4
Turkey	1	4
UK	2	7
USA	18	67
Country income status		
High	22	81
Upper-middle	4	15
Lower-middle	1	4
Study design		
Before and after	3	11
Case report	4	15
Case series	5	19
Pilot study	2	7
RCT	6	22
Qualitative	1	4
Descriptive	6	22
Decade of publication		
1970	4	15
1980	1	4
1990	6	22
2000	7	26
2010	7	26
2020	2	7
Source		
Journal	23	85
Book	2	7
Thesis	2	7
Mental health domain		
Psychological well-being	18	67
Behavioural problems	14	52
Sample size		
≤10	9	33
11–20	4	15
21–50	4	15
51–100	2	7
>100	2	7
N/A	5	19
Risk of bias		
Low	7	26
Medium	10	37
High	4	15
N/A (description only)	6	22

RCT, randomised controlled trial; N/A, not applicable.

Eighteen (67%) provide information on support focused on psychological well-being, and related domains. Of these, four (15%) targeted protective psychological factors (such as resilience and emotion management), one (4%) social anxiety disorder, one (4%) substance misuse disorder and one (4%) obsessive–compulsive disorder. Fourteen (52%) of the 27 included articles focused support on behavioural problems, such as conduct disorder. Some articles focused on both psychological well-being and behavioural problems and the percentages presented therefore total greater than 100%.

Twenty (74%) of the interventions included treatment options, 17 (63%) promotion activities and 7 (26%) prevention techniques. Eight (30%) interventions were therapeutic in nature, nine (33%) were a structured training programme, two (7%) based on physical exercise, and one (4%) medical. Seven (26%) others were intervention packages, comprised of multiple single interventions.

Eighteen (67%) described activities delivered in schools, nine (33%) of which were special schools, three (11%) special education classrooms in a mainstream school and one (4%) a mainstream school. Four (15%) were conducted in residential settings, with schooling on site. Six (22%) were conducted in healthcare facilities, one (4%) a community setting, and two (7%) artificial/research settings. One (4%) study did not provide information on the setting.

Nine (33%) of the included articles presented a case report or case series, six (22%) conducted randomised controlled trials, five (19%) used quasi-experimental methods (of which two were pilot studies) and one (4%) qualitative. Six (22%) of the articles described an intervention or support structure, without any research methods applied. One-third (33%) of articles included a sample size of less than ten participants, with only two studies (7%) including more than 100 participants.

Summarising the ages and degree of hearing loss among participants included presented a challenge. Studies provided various classifications on the severity of hearing loss, and we have converted these under the same terms in Table 2. The ages of participants and severity of hearing loss overlapped across studies, and summary statistics were difficult to present with much coherence.

**Table 2:** Individual article characteristics

First author (year)	Country	Mental health domain	Study design	Control group	Age, years	Degree of hearing loss	Participants (male/female)	Risk of bias
Ahmadi et al (2017)^20^	Iran	Social anxiety disorder	RCT	Y	12–16	Moderate to profound	48	Medium
Altshuler & Spady (1978)^21^	USA	Behavioural problems	Case series	N	4–10	Profound	3 (2/1)	Medium
Anonymous (1978)^22^	USA	Psychological well-being	Descriptive^a^	N	12+	Moderate to profound	N/A	N/A
Ashori & Najafi (2021)^23^	Iran	Emotional regulation	RCT	Y	15–19	Mild to severe	34 (0/34)	Medium
Bernstein & Denno (2005)^24^	USA	Obsessive–compulsive disorder	Case report	N	3–21	Mild to profound	1 (0/1)	Low
Boham & Selkowitz (1981)^25^	USA	Behavioural problems	Descriptive	N	6–19	Moderate to profound	N/A	N/A
Borowiec et al (2019)^26^	Poland	Self-esteem	RCT	Y	9–13	Severe to profound	28 (16/12)	Medium
Burnes et al (1992)^27^	USA	Behavioural problems	Descriptive	N	Not stated	Moderate to profound	N/A	N/A
Chapel (2005)^28^	USA	Psychological well-being and behavioural problems	Case series	N	Not stated	Moderate to profound	4 (3/1)	Low
Donovan (2003)^29^	USA	Behavioural problems	Case series	N	4–7	Moderate to profound	3 (2/1)	Medium
Dursun et al (2015)^30^	Turkey	Psychological well-being and behavioural problems	Before and after	N	8–16	Not stated	40 (24/16)	Medium
Elkayam & English (2003)^31^	USA	Psychological well-being	Pilot study	N	12–18	Mild to severe	15	Medium
Forehand et al (1974)^32^	USA	Behavioural problems	Case report	N	7	Moderate to severe	1 (1/0)	Medium
Garcia & Turk (2007)^33^	UK	Behavioural problems	Case report	N	10	Profound	1 (1/0)	Low
Greenberg & Kusché (1998)^34^	USA	Behavioural problems	RCT	Y	6–12	Severe to profound	57 (27/30)	Low
Hatamizadeh et al (2020)^35^	Iran	Resilience and behavioural problems	RCT	Y	12–15	Mild to severe	122 (74/48)	Low
Johnson & Sandberg (1992)^36^	USA	Substance abuse disorder	Descriptive	N	11–12	Mild to profound	N/A	N/A
Lasanen et al (2019)^37^	Finland	Psychological well-being	Qualitative	N	7–17	Mild to severe	16 (4/12)	Low
Nehra et al (2001)^38^	India	Psychological well-being	Before and after	Y	15–19	Moderate to profound	14	Medium
Osborne (1977)^39^	USA	Behavioural problems	Before and after	N	2–21	Mild to profound	280	High
Sarti (1993)^40^	USA	Psychological well-being	Case series	N	10–13	Mild to profound	5 (4/1)	High
Shinn (2013)^41^	USA	Behavioural problems	Case report	N	9	Moderate	1 (1/0)	Low
Sullivan et al (1992)^42^	USA	Behavioural problems	RCT	Y	12–16	Mild to profound	71 (51/21)	Medium
Tinsley & Jedlicka (2012)^43^	USA	Psychological well-being	Pilot study	N	7–19	Not stated	20	High
Troester (1996)^44^	USA	Psychological well-being, resilience and behavioural problems	Case series	N	Not stated	Moderate to profound	3 (3/0)	High
Vreeland & Tourangeau (2003)^45^	USA	Psychological well-being	Descriptive	N	8–21	Mild to profound	N/A	N/A
Wright et al (2012)^46^	UK	Psychological well-being	Descriptive	N	8–18	Not stated	N/A	N/A

RCT, randomised controlled trial; Y, yes; N, no; N/A, not applicable.

a. Description of an intervention with no research methods applied.

### Quality assessment

The six (22%) articles that describe (rather than evaluate) an intervention were not included in the quality assessment. Of the 21 remaining, 7 (33%) were rated as having a low risk of bias, 10 (48%) medium and 4 (19%) high. Potential sources of bias among those rated to have a medium or high risk, included: inappropriate statistical analysis, including the absence of statistical power or sample size calculations in RCTs; no clinical information provided for participants in a case series; and unsuitable measurement of outcomes, including collection time points and screening method.

### Types of support

#### Psychological well-being

As seen in Supplementary Table 1, 17 articles targeted psychological well-being and related factors (for example resilience).

Five (29%) of these focused on forms of counselling, psychotherapy or group support.^28,31,37,43,44^ Examples included peer-support groups and therapeutic play. Four (24%) provided structured training and skills-based programmes, including group assertiveness training for social anxiety disorder.^20,23,35,36^ Two (12%) were focused on physical exercise and related activities, including ice-skating and dance lessons.^26,30^ One (6%) study investigated the impact of hearing aids on psychological well-being.^38^

The remaining five (29%) articles provided information on intervention ‘packages’ that included more than one intervention approach.^22,24,40,45,46^ These were typically delivered in healthcare or residential settings. Individual interventions described in the packages were typical of those provided in mental health programmes for the general population, and included cognitive–behavioural therapy, medication, art therapy and peer-support groups.

#### Behavioural problems

Fourteen articles provided support for behavioural problems. In four articles, the intervention or support provided addressed both psychological well-being and behavioural problems.

Five of the 14 (36%) provided counselling or variations of psychotherapy, including video-counselling.^25,28,39,42,44^ Three (21%) provided parents with training, and typically included skills-building exercises, using role-play and similar techniques.^32,33,41^ Three (21%) focused training efforts on children.^29,34,35^ For example, the PATHS (Promoting Alternative Thinking Strategies) curriculum, which includes daily activities at school, delivered by teachers.^34^ One (7%) supported physical activity, in this instance, ice-skating.^30^ A further two (14%) articles described a support package including various activities.^21,27^ This included Walden House, a residential programme for children with hearing loss and behavioural problems, providing problem-solving skills, family therapy and role-play exercises.^27^

### Theoretical underpinnings

Twenty three of the 27 articles provided information on the theory or evidence used to develop the intervention. The remaining four articles provided descriptions of logic-based arguments for the intervention model, without substantial information on evidence or theory. Of the 23 articles providing this information, 12 (52%) provided evidence of previous use or effectiveness of the intervention among children without disabilities or hearing loss, four provided (17%) evidence of its use with children with and without hearing loss, and four (17%) articles provided evidence of the intervention among children with hearing loss only. Four (17%) based the intervention on theories developed for children without hearing loss, such as the ABCD (Affective-Behaviour-Cognitive-Dynamic) model of child development.

### Adaptations made to interventions

Of the 27 articles, 16 described specific considerations and adaptations made to the intervention to accommodate the needs of children with hearing loss, particularly when the support provided was initially developed for the general population.

Of these, 13 (81%) described considerations of communication methods and needs, such as reading ability. Nine (56%) used sign language or provided sign language interpretation. Three (19%) made adaptations to the physical environment in which the intervention was delivered. For instance, seating groups in a circle to maximise visual contact, or providing pictures and posters to facilitate understanding. Three (19%) adapted the core structure, components, tools and methods of established interventions. For example, established programmes for the general population, such as D.A.R.E. and the PATHS curriculum were adapted and prepared with support from hearing loss specialists.

### Outcomes

#### Effectiveness

In total, 19 articles provided information on the effectiveness of the intervention or programme.

Eleven of these presented outcomes with regards to psychological well-being, with all but one finding some evidence of a positive impact of the intervention. Specifically, three (27%) provided evidence on the effectiveness of training programmes for children with hearing loss, including resilience training. One of these, targeting social anxiety disorder, reported no significant differences in symptomology between intervention and control groups in children with profound hearing loss, but did see a difference for those with moderate hearing loss. Two (18%) demonstrated the positive effect of physical activity interventions; dance lessons and ice-skating. Therapy-based interventions were found beneficial by two (18%) studies, one of which, a qualitative study, provided evidence of the positive perceived benefit from participants of regular peer-support groups, although changes in well-being were not measured nor observed with other standardised methods. One (9%) other found no difference in participants pre- and post-intervention after receiving child-centred play therapy. One demonstrated significant improvements in well-being, including reduced symptoms of anxiety and depression, after children had used hearing aids for 6 months. Two studies (18%) provided evidence on the effectiveness of intervention packages comprised of various approaches, including a package for obsessive–compulsive disorder. Of those described, five (45%) demonstrated improvements in scores on quantitative standardised measurement tools (such as the Strengths and Difficulties Questionnaire) after the intervention was implemented, of which four (36%) RCTs showed significant improvement in the intervention group and not the control group.

With regards to behavioural problems, 11 studies reported on outcomes, of which all but one found some evidence of a positive impact of the intervention. Five (45%) found evidence on the benefit of training programmes, including parent behaviour training and teacher delivered resilience behaviour training for children. Three (27%) demonstrated the positive impact of therapy-based interventions on behaviour, although one (9%) showed no benefit. One study (9%) showed improvements among children on the Strengths and Difficulties Questionnaire, including the behavioural domain, after regular ice-skating sessions. One (9%) showed the benefit of a triadic intervention model for behavioural problems, focused on a core of reinforcement in schools. Seven (64%) reported positive improvements in behavioural outcomes, assessed through standardised measurement tools. Three RCTs (27%) demonstrated significant improvement in the intervention group and not the control, after delivery.

#### Other outcomes

Four articles provided evidence about the acceptability of the intervention to participants, delivery agents and/or parents. In one pilot study, this was the primary outcome that was measured quantitatively by questionnaire, with participants reporting good acceptance of the counselling method provided to support psychological well-being. In another, teachers reported feeling motivated to deliver the functional communication training for behavioural problems, and were happy with the training provided. In the third, both parents and delivery agents (clinicians) provided positive feedback in a directed workshop, with regards to the structure and components of the Webster-Stratton programme for behavioural problems. In the final case study of parent–child interaction therapy for behavioural problem, the mother expressed the value of learning to play with her child and manage their behaviour in a way that was fun and engaging for them.

## Discussion

### Summary of evidence

To the best of our knowledge, this is the first attempt to systematically identify and map the evidence on mental health support for children and adolescents with hearing loss. The scoping review identified 27 articles, two-thirds of which were conducted in the USA and two-thirds published before 2010. A number of interventions and support initiatives were described in the literature, including a broad range of therapy and counselling, and targeted training programmes, for both children and caregivers.

The interventions identified were, by and large, found to be effective at supporting the mental health and well-being of children with hearing loss. Although not all articles provided information on outcomes, nor all via quantitative assessment, those that did offered some encouraging signs as to the benefits of mental health support for children with hearing loss. Dance lessons, resilience training programmes and hearing aids demonstrate the diverse gamut of effective interventions that can be utilised to support children with hearing loss. Interventions provided treatment, prevention and promotion options in various settings, supporting recent advocacy for population- and community-level support for child mental health.^47^ As the evidence suggests, an effective and sustainable approach to child mental health includes support from an early age, improving information and awareness, providing social and emotional learning activities, increasing detection and identification, and improving access to treatment and rehabilitation. The interventions in this review provide some examples of interventions that may be effective within such a system, although as we will go on to discuss, the strength of the current evidence is limited.

Most of the interventions identified in this review were provided in school, which follows evidence from among the general population as to the benefits of integrated mental health provision within education systems.^48^ Interventions at school level provide a continuum of care that benefits a child's mental health, as well as their educational attainment. Only one identified intervention was conducted within a mainstream classroom, despite the recent global movement towards inclusive education.^49^ That said, a recent report outlines the limited transformations in education systems across the world, with most countries facing difficulties in inclusive provision, with understanding of what this means, and resources and evidence on good practice variable across regions.^50^ As reform continues to spread, there will need to be evidence gathered on appropriate mental health interventions in inclusive classrooms, to ensure children with hearing loss are not excluded.

When considering interventions for children with hearing loss, it is important to note components and delivery methods unique to this group. Many of the interventions were initially developed for use in the general population, and more than half of the included articles described interventions that had been developed from evidence for children without hearing loss and adapted for the needs of children with hearing loss. Adaptations were most commonly focused on communication, and often sign language. Communication is often cited as a major barrier and facilitator to accessing healthcare for people with hearing loss, and it is appropriate to see this as the focus of many adaptations.^51^ In some settings (typically high-income settings) such as the UK, and as seen in the included article from Wright et al (2012), specialist mental health services exist for children with hearing loss, where sign language and other accommodations are embedded in the service provided.^46^ However, in other settings, provision of communication technologies and sign language interpretation may not always be possible, given restricted resources. Often, and especially in low- and middle-income countries, mental health interventions are adapted from one context to another, in order to meet the culture, needs and resources of a population. In low- and middle-income countries, this often includes adapting interventions developed in high-income settings, although there are good examples of adaptation in the other direction, in which we see novel and effective mental health interventions developed in low-resource settings and adapted to those in high-income settings (an example being the Friendship Bench, developed in Zimbabwe and adapted to New York City in the USA).^52^ Adaptation is common and needed, and the well-evidenced stages of this process should be noted by those looking to adapt mental health interventions for children with hearing loss, in order to ensure appropriate intervention components and delivery mechanisms. This will include standard adaptations to the context, but also specific adaptation to the needs of children with hearing loss, such as those seen in this review. We did not find any studies that provided evidence of an adaptation process through formative research, theory of change workshop, feasibility study, or similar methods, and this would be a useful area of research going forwards, to promote interventions for children with hearing loss that are contextually and culturally appropriate, and hopefully then effective and sustainable. These approaches also promote the participation of stakeholders, including those with hearing loss and/or mental health conditions.

### Evidence gap

Although 27 articles were identified, there is concern about the breadth, scope and strength of the evidence within the literature available. With two-thirds (67%) of articles published before 2010, and 41% pre-2000, it is evident that the published literature on this topic is not growing at a fast rate. Interest and research in disability has steadily increased since the United Nations Convention on the Rights of Persons with Disabilities (2008), and calls for further evidence on scalable mental health interventions have been delivered by leading figures for many years.^53^ Despite an increased focus on global mental health and disability rights, this review suggests that mental health support for children with hearing loss is not gaining sufficient interest in the research community. The Lancet Commission on Global Mental Health and Sustainable Development, published in 2018, may stimulate additional research in the future, calling as it does for a focus on culturally appropriate and participatory approaches to translating evidence for promotion and care in mental health across diverse populations.^54^ Our approach to classification using a continuum from well-being to mental health diagnosis draws on these ideas. Exploring the evidence among adult populations is also needed given the higher prevalence of hearing loss in this age group.

Furthermore, nearly all (81%) of the included articles come from high-income settings, with two-thirds (67%) conducted in the USA. Just one (4%) was conducted in a lower-middle or low-income setting. There are no articles from Latin America or Africa, and just one study from Asia. The geographical scope of study into appropriate interventions for children with hearing loss must increase. Given that 80% of the world's population and people with disabilities are living in low- and middle-income countries, there is an urgent need for evidence on contextualised interventions.^55^ Relying on data from high-income settings has caused concern in the field of global mental health, as doing so reduces long-term effectiveness and sustainability of mental health interventions, especially when biomedical models of care are transferred to settings where they may not be culturally accepted, contextually feasible or appropriate.^54^ There is growing evidence on new and adapted mental health interventions for the general population in low- and middle-income settings, and as shown in this review, there is the possibility of adapting these to suit the needs of children with hearing loss (for example sign language provision). Evidence is needed on the process of adaptation and contextualisation, in order to inform delivery in the given country, and to provide a research framework for others.

Most of the studies were assessed to have medium or high risk of bias. Just six (22%) of the studies available and included are RCTs. The majority of included articles offer interesting case reports, but these provide the rationale for larger-scale trials, rather than high-quality evidence in and of themselves. Most of the studies included have also been conducted with very small sample sizes. Nearly half (48%) of studies contained a sample size smaller than 20, increasing to two-thirds (67%) when including those articles with no sample size available. To improve the evidence base with which to stimulate service provision and policy, there needs to be experimental research, with large samples, with which to build confident conclusions and inform scalable interventions. Potential sources of bias must be addressed, including appropriate statistical methods, including sample size calculations for sufficient power.

### Limitations

There are several limitations to this review. First, we excluded articles that were not in the English language, and there may well be evidence missed that has been published in other languages. This is particularly important when considering the limited evidence from South America and East Asia. Our age range, 6–18 years, is broad and although not necessarily a limitation, it is important that readers of this review pay close attention to the targeted age range of interventions of interest. Younger children and older adolescents will respond differently to interventions, and will have different mental health needs, and not all interventions will be applicable across age groups. Second, we could not source 19 full-text articles, in which there may have been relevant and included studies. We contacted authors directly and utilised various institutional access agreements, but still could not find these for assessment. Third, we did not assess publication bias. When interpreting the results on effectiveness, readers should be conscious of the risk of positive publication bias and a potential lack of interest to publish results on ineffective interventions. Finally, in their framework for scoping reviews, Arksey & O'Malley recommend an optional sixth step; consultation with stakeholders.^18^ Such consultation is time-consuming and costly, and was not feasible when undertaking this study, and may be an important area to address in any future and updated reviews.

### Implications

This review has identified a number of articles and interventions, which generally showed positive results, but there are concerns over the breadth of the evidence available. There is evidence of some effective interventions, such as dance lessons and resilience training, but the applicability of the available evidence is limited by geographic location and publication date, and there is a need for more studies applying high-quality research methods. To improve the current evidence base, we must strengthen the quality of the research methods used and provide further research from low- and middle-income countries on adapting interventions to local contexts and on interventions tested at scale. Having a community of researchers set future research priorities, as informed by practitioners and people affected, will focus and strengthen research activities going forwards.

### Recommendations on mental health support for children with hearing loss

Based on the findings of this review, we have listed below a number of considerations for delivering mental health support for children with hearing loss, including adaptations to existing mental health interventions.

- Consider the intervention models identified in this review, including the following.
  - Peer-support: children with hearing loss can benefit from interaction with peers, reducing isolation, while helping them learn more about themselves and their experience as a child with hearing loss. Peer-support can act as both a treatment and health promotion option.
  - Resilience training: identified by a number of studies in this review, resilience training may be an effective way of preventing mental health conditions, especially if implemented at an early age. This type of training may be particularly appropriate to integrate into schools.
  - Emotional and behavioural management training: this type of structured training programme can be provided for both parents and children. Helping parents learn the tools with which to support their child's emotional regulation and behaviour may help create a home environment that promotes well-being and positive coping strategies. Training children themselves, possibly in school, will further help children with hearing loss build the toolkit with which to understand and approach the challenges they face.
  - Physical activity: physical activity is a simple way to build well-being and promote mental health. In this review, the physical interventions identified were specialist in nature – ice-skating and dance therapy are not necessarily available in all settings – but adapting these techniques and principles to a different context may prove effective.
- Consider the following when adapting or developing an intervention.
  - Knowledge and awareness: it is important that the delivery agent is given appropriate training and sensitisation on disability and hearing loss. There may also be cause to provide information to children without hearing loss in the same setting, in order to reduce any apparent stigma or discrimination; for example, to hearing children in a mainstream classroom.
  - Communication methods: each child will have different communication needs, depending on their severity of hearing loss and personal preferences. Include appropriate training for delivery agents on communication methods, including clarity of speech and the language used. Where necessary, provide sign language interpretation, and where relevant provide training on hearing aid or cochlear implant use, as this knowledge will facilitate improved communication and reduce stigma.
  - Physical environment: consider the environment in which the intervention is delivered. This may be a mainstream classroom, for instance, that is not set up to accommodate the needs of children with hearing loss. Considering the configuration of seating in a group is one example; children sitting in a circle may help improve visual contact and support those who lipread. Keeping the environment quiet and free from distractions will also help children with hearing loss, as might keeping the door closed and adding soft material to the underside of chairs.
  - Intervention materials and techniques: where suitable, use visual aids and cues to support the intervention components. One example may include pictures matched to emotions, to aid the understanding and communication of feelings. Other techniques to reinforce behaviours and actions may include role-play, storytelling and peer-feedback.
- Consider the following during intervention development and implementation.
  - Interventions identified in this review benefitted from clear communication and coordination between different groups involved. For instance, interventions in schools were strengthened when teachers and parents worked together.
  - Conduct feasibility studies and pilot studies, where possible, in order to make any necessary amendments early in the implementation process. This will also help inform others in the future.
  - Providing a range of interventions within a service or facility (such as a school), where this is feasible, may provide the best model with which to support a personalised approach for each child.
- Consultation and coordination may help promote feasible, acceptable and sustainable intervention programmes.
  - Consult the children, their parents, mental health and hearing specialists, and the delivery agents (such as teachers). Understand their needs and context in the adaptation or development phase.
  - In all, talk to the children who are the intended target of the intervention about their own needs and preferences for intervention components, including communication methods.

## Data Availability

Data availability is not applicable to this article as no new data were created or analysed in this study.

## Supplementary material

For supplementary material accompanying this paper visit http://dx.doi.org/10.1192/bjo.2021.1045. click here to view supplementary material
