# Supplementary material for: Mental health support for children and adolescents with hearing loss: scoping review
Source: BJPsych Open. 2021 Dec 6;8(1):e9. doi: 10.1192/bjo.2021.1045 (PMC8693903; doi:10.1192/bjo.2021.1045)
Supplement: Supplementary file 1 [file S2056472421010450sup001.docx]

**Supplementary File 1. Example search strategy – MEDLINE April 2020**

Database: Ovid MEDLINE(R) <1946 to April Week 4 2020>

Search Strategy:

--------------------------------------------------------------------------------

1 child/ (1670976)

2 minors/ (2563)

3 child*.ti,ab. (1214777)

4 kid*.ti,ab. (414687)

5 minor*.ti,ab. (255405)

6 (boy* or girl*).ti,ab. (208080)

7 Adolescent/ (2005972)

8 adolescen*.ti,ab. (232901)

9 (preadolescen* or pre-adolescen*).ti,ab. (3678)

10 teenage*.ti,ab. (18627)

11 (teen or teens or teener).ti,ab. (9132)

12 (preteen* or pre-teen*).ti,ab. (414)

13 pubescent*.ti,ab. (723)

14 (prepubescent* or pre-pubescent*).ti,ab. (972)

15 (youth* or youngster* or young people* or young person*).ti,ab. (83048)

16 underage*.ti,ab. (1010)

17 Students/ (57499)

18 Schools/ (37462)

19 student*.ti,ab. (229969)

20 pupil*.ti,ab. (24690)

21 schoolchild*.ti,ab. (12691)

22 ((school or college) adj3 (pupil* or student*)).ti,ab. (37391)

23 (schoolage* or (school adj1 age*)).ti,ab. (19478)

24 (highschool* or (high adj1 school*)).ti,ab. (26510)

25 (primary school* or elementary school*).ti,ab. (18519)

26 only child/ (224)

27 siblings/ (11051)

28 (sibling* or brother* or sister*).ti,ab. (84690)

29 offspring.ti,ab. (61492)

30 (("6" or "7" or "8" or "9" or "10" or "11" or "12" or "13" or "14" or "15" or "16" or "17" or "18") adj (year* old or year* of age)).ti,ab. (171080)

31 ((six or seven or eight or nine or ten or eleven or twelve or thirteen or fourteen or fifteen or sixteen or seventeen or eighteen) adj (year* old or year* of age)).ti,ab. (11320)

32 (age* adj (six or seven or eight or nine or ten or eleven or twelve or thirteen or fourteen or fifteen or sixteen or seventeen or eighteen) adj year*).ti,ab. (448)

33 (age* adj ("6" or "7" or "8" or "9" or "10" or "11" or "12" or "13" or "14" or "15" or "16" or "17" or "18") adj year*).ti,ab. (33480)

34 or/1-33 (4101900)

35 exp hearing loss/ (68491)

36 hearing disorders/ (14527)

37 (hearing adj3 (loss or impair* or disabilit* or disorder* or difficult* or handicap* or acuity or deficit* or defect* or dysfunction*)).ti,ab. (51596)

38 "loss of hearing".ti,ab. (1315)

39 "hard of hearing".ti,ab. (1197)

40 (hard adj2 hearing).ti,ab. (1197)

41 (deaf* or deafness).ti,ab. (32848)

42 exp Auditory Diseases, Central/ (2280)

43 (auditory adj3 (loss or impair* or disabilit* or disorder* or difficult* or handicap* or acuity or deficit* or defect* or dysfunction* or comprehension)).ti,ab. (5847)

44 (audiological adj3 (loss or impair* or disabilit* or disorder* or difficult* or handicap* or acuity or deficit* or defect* or dysfunction* or comprehension)).ti,ab. (142)

45 persons with hearing impairments/ (2692)

46 or/35-45 (110262)

47 Mental Health/ (37303)

48 mental health.ti,ab. (113359)

49 (mental* adj3 (disabilit* or health or distress or disorder* or ill* or disturbance or hygiene)).ti,ab. (167531)

50 Psychological Distress/ (355)

51 psychological*.ti,ab. (176844)

52 (psychological* adj3 (disabilit* or health or distress or disorder* or ill* or disturbance)).ti,ab. (28086)

53 psychosocial*.ti,ab. (79800)

54 (psychosocial* adj3 (disabilit* or health or distress or disorder* or ill* or disturbance)).ti,ab. (7707)

55 psychiatric*.ti,ab. (166884)

56 (psychiatric* adj3 (disabilit* or health or distress or disorder* or ill* or disturbance)).ti,ab. (49520)

57 emotion*.ti,ab. (160488)

58 (emotion* adj3 (disabilit* or health or distress or disorder* or ill* or disturbance)).ti,ab. (16370)

59 exp Mental Disorders/ (1225111)

60 affective symptoms/ (12866)

61 Anxiety/ (79535)

62 depression/ (116762)

63 (affective adj2 (disorder* or symptom*)).ti,ab. (17195)

64 (anxiety adj2 (disorder* or symptom* or state)).ti,ab. (45057)

65 (depress* adj2 (disorder* or syndrome* or symptom* or state)).ti,ab. (90511)

66 anxious.ti,ab. (14266)

67 depress*.ti,ab. (394544)

68 mood disorder*.ti,ab. (14229)

69 psychos*.ti,ab. (136770)

70 (psychos* adj2 (symptom* or state)).ti,ab. (3576)

71 schizophreni*.ti,ab. (107717)

72 (schizophreni* adj2 (symptom* or state)).ti,ab. (6848)

73 (bipolar or bi-polar).ti,ab. (52804)

74 ((bipolar or bi-polar) adj2 (disorder* or symptom*)).ti,ab. (26452)

75 personality disorder*.ti,ab. (17440)

76 (trauma* adj3 (disorder* or symptom* or state)).ti,ab. (16453)

77 Happiness/ (4253)

78 (wellbeing or well-being or wellness).ti,ab. (77645)

79 (happy or happiness).ti,ab. (12385)

80 mental* well*.ti,ab. (2280)

81 positive affect.ti,ab. (4359)

82 psychological distress/ (355)

83 Stress, Psychological/ (118903)

84 Resilience, Psychological/ (5455)

85 resilien*.ti,ab. (22733)

86 stress*.ti,ab. (691192)

87 loneliness/ (3587)

88 sadness/ (101)

89 lonel*.ti,ab. (5604)

90 sad*.ti,ab. (20691)

91 exp "attention deficit and disruptive behavior disorders"/ or child behavior disorders/ (50391)

92 Social Behavior Disorders/ (4748)

93 (behavio?r* adj3 (disorder* or challeng* or problem* or antisocial*)).ti,ab. (42571)

94 (conduct* adj2 (disorder* or problem*)).ti,ab. (8823)

95 or/47-94 (2503952)

96 adolescent health services/ (5535)

97 early intervention, educational/ (2987)

98 preventive health services/ (13317)

99 primary prevention/ (18267)

100 secondary prevention/ (20087)

101 tertiary prevention/ (151)

102 health promotion/ (72829)

103 health education/ (60138)

104 school health services/ (17065)

105 school mental health services/ (12)

106 exp mental health services/ (94550)

107 health services for persons with disabilities/ (106)

108 interven*.ti,ab. (842352)

109 program*.ti,ab. (731547)

110 pilot.ti,ab. (125392)

111 remed*.ti,ab. (39626)

112 support*.ti,ab. (1273741)

113 promot*.ti,ab. (847804)

114 prevent*.ti,ab. (1213166)

115 or/96-114 (4344591)

116 34 and 46 and 95 and 115 (1856)

**Supplementary File 2. List of organisations manually searched for grey literature**

1. Able Child Africa
2. Action on Hearing Loss
3. ADD International
4. Atfaluna Society for Deaf Children
5. Caudwell Children
6. CBM
7. Deaf Child Worldwide
8. Deafblind International
9. DeafKidz International
10. European Federation of Hard of Hearing People
11. Global Coalition of Parents of Children who are Deaf or Hard of Hearing
12. Global Foundation for Children with Hearing Loss
13. Hear the World Foundation
14. Hearing Health Foundation
15. Hearing International
16. Hearing Link
17. Humanity & Inclusion
18. International Federation of Hard of Hearing People
19. International Federation of Hard of Hearing Young People
20. Leonard Cheshire
21. Light for the World
22. National Deaf Children’s Society
23. Quota International
24. Sense International
25. Sightsavers
26. SignHealth
27. Sound Seekers
28. Starkey Hearing Foundation
29. Vaani
30. World Federation of the Deaf
31. World Wide Hearing

**Supplementary Table 1. Intervention characteristics and outcomes**

| First author (year), country | Study design | Mental health domain | Type of support & target group | Setting | Intervention description | Theoretical underpinning | Key findings |
| --- | --- | --- | --- | --- | --- | --- | --- |
| *Psychological wellbeing* | | | | | | | |
| Ahmadi (2017), Iran | Randomised controlled trial (RCT) | Social anxiety disorder | Treatment  Children with hearing loss | School (special) | Group assertiveness training, provided over 10 sessions, lasting 10–60 min. Activities are aligned to: rejecting a request; expressing one’s own limitations; requesting; initiating socialisation; expressing positive feelings; embracing criticism; being assertive in situations where one should help and receives negative feedback | Previous evidence on the effectiveness of assertiveness training for social anxiety disorder among children and adolescents without disabilities | No significant difference between intervention and control for adolescents with profound hearing loss; Social Anxiety Disorder, and sub-scales fear and avoidance, significantly lower in adolescents with partial hearing loss in intervention group, compared to the control |
| Anonymous (1978), USA | Descriptive | Psychological wellbeing | Treatment, promotion  Children and adults with hearing loss | Hospital | Description of the mental health services provided at a tertiary hospital, including: occupational therapy; art therapy (painting, leatherworking); psychodrama; dance therapy; group psychotherapy; recreational activities (roller-blade, films, social functions) | None stated | N/A (description only) |
| Ashori  (2021), Iran | Randomised controlled trial (RCT) | Emotion regulation | Promotion, prevention  Children with hearing loss | School (special) | Emotion management strategy training delivered in schools, focused on: awareness of emotions and feelings; emotion understanding skills; situation selection; prevention-related skills; cognitive assessment; response moderation | Previous evidence on the intervention used with children without disabilities | Significant difference between intervention and control groups. Intervention group showed improvements in adaptive and non-adaptive emotional regulation strategies, as well as improved life orientation. The control group did not. |
| Bernstein (2005), USA | Case report | Obsessive-compulsive disorder (OCD) | Treatment  Children with hearing loss | School (special) | Various treatment options delivered by teachers described, including: redirecting compulsive behaviours; teaching internal controls; ‘OCD time’ for behavioural to occur without redirection; approach and resist therapy; medication | None stated | Case report of Susan’, who saw fewer intervals of withdrawal or agitation. Her mood and affect improved from medication. OCD frequency decreased |
| Borowiec (2019), Poland | Randomised controlled trial (RCT) | Self-esteem | Promotion  Children with hearing loss | School (special) | Dance lessons held at school with a trained instructor in scheduled physical education sessions. Students wear Audiva High Pitch Training System vibrating headphones. Classes last 40 min and are held twice per week | Previous evidence on the positives of dance lessons on physical and mental health among those with and without hearing loss. Vibrations from music shown to activate the somatosensory part of the cerebral cortex | There was a significant difference between intervention and control groups at post-test, with children in the intervention group presenting higher self-esteem than controls. In both groups, self-esteem significantly improved from pre- and post-test timepoints |
| Elkayam (2003), USA | Pilot study | Psychological wellbeing | Treatment  Children with hearing loss | Audiology service | Counselling delivered by an educational audiologist, facilitated by self-assessment questionnaires | Previous evidence on the benefit of questionnaires to facilitate counselling sessions with children without disabilities | Mean scores indicated a neutral opinion to follow-up questions on the benefits of the counselling, although individual perceptions varied. Learning new information about their hearing loss was the most commonly reported benefit among the adolescents. Adolescents reported positively with regards to acceptability of the counselling. |
| Johnson (1992), USA | Descriptive | Substance abuse disorder | Prevention  Children with hearing loss | School (special) | D.A.R.E programme, provided over 17 weeks in school. Topics included in lessons: drug use and misuse; resisting pressure to use drugs; resistance techniques; building self-esteem; managing stress without drugs; role-modelling; support systems | The programme has been widely implemented across mainstream schools in the USA since its inception in 1983 | N/A (description only) |
| Lasanen (2019), Finland | Qualitative | Psychological wellbeing | Prevention, promotion  Children with hearing loss | Not stated | Peer-support groups | Previous evidence into the positive effect of peer-support interventions for children and adults with and without disabilities | The qualitative research identified various benefits of the peer-support groups, including: social peer support (gaining new friends and community); cognitive peer support (sharing information about hearing loss, hearing aids, cochlear implants); functional peer support (training on coping strategies, hearing aid maintenance); emotional peer support (support from the community to build up trust, joy, courage, comfort) |
| Nehra  (2001), India | Before and after | Psychological wellbeing | Treatment  Children with hearing loss | Health | Introduction of hearing aids | Previous evidence on the negative impact of poor communication and hearing on mental health and wellbeing, and previous evidence of the positive impact of hearing aids on psychosocial dysfunction | Six months after receiving a hearing aid, participants showed significant improvement in psychosocial dysfunction, depression, anxiety and wellbeing. State anxiety increased in the control group and group with severe hearing loss, but decreased in those with moderate hearing loss. Feelings of helplessness only reduced in those with moderate hearing loss |
| Sarti  (1993), USA | Case series | Psychological wellbeing | Treatment, promotion  Children with hearing loss | School (special) | Various interventions described within at a residential school, including: group therapy; role-play on positive emotions and behavioural; rap; videotaped session review | Therapeutic community model based on developmental psychology, therapeutic community and social psychology | Observation indicates improved psychological wellbeing, including improved self-concept and ego control. Teachers reported improved relationship with their students |
| Tinsley  (2012), USA | Pilot study | Psychological wellbeing | Treatment  Children with hearing loss and families | Mental health service | Equine assisted psychotherapy, delivered by a social worker and equine specialist over 10 one-hour sessions. The intervention supported parents with hearing loss and hearing children, as well as hearing parents and children with hearing loss. | Previous evidence of equine assisted counselling in use with youth without disabilities | Administrative challenges meant post treatment data was not systematically collected and insufficient data was available. Anecdotally, feedback was positive, with one family seeing no benefit |
| Vreeland (2003), USA | Descriptive | Psychological wellbeing | Treatment, prevention, promotion  Children with hearing loss | Residential, school | Various described within a residential setting, including: psychodynamic therapy; psychoeducation; play therapy; group peer-support and therapy | Previous evidence on the needs of and treatment options for children with hearing loss, based on literature and implementation experience | N/A (description only) |
| Wright  (2012), UK | Descriptive | Psychological wellbeing | Treatment  Children with hearing loss | Mental health service | Description of the packages provided in deaf child and adolescent mental health services, comprised of various therapies, such as cognitive-behavioural therapy | Deaf mental health services developed across the UK since 1963 | N/A (description only) |
| *Psychological wellbeing and behavioural problems* | | | | | | | |
| Chapel  (2005), USA | Case series | Psychological wellbeing, behavioural problems | Treatment  Children with hearing loss | School (special) | Child-centred play therapy, provided daily for 35-minutes. | Theories and evidence on the positive benefits of child-centred play therapy for children without disabilities | Pre- and post-treatment measures of the participants did not provide evidence of improvement in the participants |
| Dursun (2015), Turkey | Before and after | Psychological wellbeing, behavioural problems | Treatment, promotion  Children with hearing loss | Community ice-skating centre | Ice-skating with an instructor for one hour, twice per week, for three months | Previous evidence on the positive benefits of physical activity for wellbeing among children with disabilities | Significant improvement seen in scores on the Strengths and Difficulties Questionnaire, including emotional, behavioural and stress. Improvement also seen in self-concept and the impact of psychiatric symptoms on their lives |
| Hatamizadeh (2020), Iran | Randomised controlled trial (RCT) | Resilience, behavioural problems | Promotion  Children with hearing loss | School (mainstream) | Teacher delivered resilience training, provided twice per week, for six weeks. Sessions targeted the integration of emotions, feelings, and behaviour to promote resilience, and included: group and individual activities (telling stories, drawing); group discussion | Intervention based on evidenced programmes for children without disabilities, including: PATHS Curriculum; Penn Resiliency Program; Aussie Optimism Resilience Skills Program | Resilience scores increased in the intervention group and were significantly higher than the control. Effect size was large. Children in the intervention group also saw improved scores related to hyperactivity, emotional symptoms, conduct, peer problems and prosocial behavioural |
| Troester (1996), USA | Case series | Psychological wellbeing, resilience, behavioural problems | Treatment, promotion  Children with hearing loss | School (special classroom in mainstream) | Therapeutic play group delivered in schools, focused on: communication; behavioural; skill-building | Previous evidence on the positive effect of therapeutic play therapy for children without disabilities | The three boys demonstrated improvement psychosocial development and wellbeing, including resilience, assertiveness, social skills and few disruptive behaviours |
| *Behavioural problems* | | | | | | | |
| Altshuler (1978), USA | Case series | Behavioural problems | Treatment, promotion  Children with hearing loss and parents | Residential, school | Various approaches described including: support from a psychologist, medication, parent training, safe/quiet spaces in classroom, structured routines | None stated | The three children demonstrated fewer tantrums, fewer compulsive behaviours, better engagement with others and reduced symptoms of anxiety |
| Boham  (1981), USA | Descriptive | Behavioural problems | Treatment, promotion  Children with hearing loss | School (special) | Video counselling, delivered by teachers and psychologists. Sessions include: role-modelling; calming techniques; learning appropriate responses to certain stimuli | None stated | N/A (description only) |
| Burnes  (1992), USA | Descriptive | Behavioural problems | Treatment, prevention  Children with hearing loss and families | Residential, school | Description of a programme for Deaf children at Walden House, a residential and education setting. The behavioural programme includes: ‘Group processing’ with peer-feedback; problem-solving skills; spoken language skills; American Sign Language training; role-play; family therapy | None stated | N/A (description only) |
| Donovan (2003), USA | Case series | Behavioural problems | Treatment Children with hearing loss | School (special classroom in mainstream) | Functional communication training, delivered by teachers, which includes: observing and identifying challenging behavioural; ignoring these behaviours; reinforcement of desired behaviours | The author conducted a literature review of functioning communication training for children with and without disabilities exhibiting challenging behaviour | For all three participants, there was a decrease in challenging behavioural and increased use of trained communicative behavioural. Teachers were motivated and happy with the intervention and training |
| Forehand (1974), USA | Case report | Behavioural problems | Treatment Parents | Artificial research setting | Parent behavioural training, delivered by a therapist, across 13 sessions. These included: instruction on the use of reinforcement during play; modelling reinforcement techniques; role-play with the parent; practice with live in-ear feedback from the therapist | Previous evidence of positive benefits among children without disabilities | The single participant’s behavioural rating scores improved after treatment, with fewer problem behaviours shown |
| Garcia  (2007), UK | Case report | Behavioural problems | Treatment Parents | Health clinic | Webster-Stratton programme, including 13 sessions for parent groups, held once per week, lasting two hours. The programme included: video clips of parents and children interacting; psychoeducational handouts; role-play; communication training | Previous evidence of extensive use among children without disabilities | Results for one child presented. He showed improved scores in the behavioural measure, with a reduction in the problem score and intensity. Parenting stress and distress reduced by 30%. Both parents and clinicians reported positive experiences |
| Greenberg (1998), USA | Randomised controlled trial (RCT) | Behavioural problems | Prevention, promotion  Children with hearing loss | School (special classroom in mainstream) | PATHS curriculum, delivered daily in school for 22 weeks. The programme includes: training in self-control through role-play; psychoeducation and emotional literacy; cognitive problem-solving | Based on the ABCD (Affective-Behaviour-Cognitive-Dynamic) model of child development | After year 1, children in the intervention group saw improvement in self-image, emotional adjustment and social competence. However, there was no significant improvement in behavioural problems. The results were replicated with the control group, who were given the intervention via waiting list in year 2, although they saw a greater reduction in behavioural problems, due to improved teacher experience and more sessions |
| Osbourne (1977), USA | Before and after | Behavioural problems | Treatment, prevention  Children with hearing loss | School (special) | Triadic intervention model, provided in school classrooms by teachers and a psychologist. Methods to improve behavioural focused on reinforcement | The triadic intervention model, comprising the target, mediator and consultant, is directly applied | Two-thirds of participants displayed improved behaviour over the 6-year observation period |
| Shinn  (2013), USA | Case report | Behavioural problems | Treatment  Parents | Artificial research setting | Parent-child interaction therapy, delivered by a therapist for parents to implement at home. Sessions included: communication training; behavioural intervention (reinforcement, punishment); parent skills building in managing behavioural and compliance | Previous evidence on the positive benefits of parent-child interaction therapy for children without disabilities | Scores on the behavioural measures improved and parenting stress decreased in a single subject study. The child’s mother reported on the benefits of the intervention in helping manage her child’s behavioural |
| Sullivan (1992), USA | Randomised controlled trial (RCT) | Behavioural problems | Treatment  Children with hearing loss (who had experienced sexual abuse) | Residential, school | Psychotherapy, delivered once per week for 36 weeks | Authors conducted a literature review on therapeutic interventions for sexually abused children without disabilities | Those in intervention arm had significantly fewer behavioural problems one year after implementation. This was true for girls and boys, with the intervention benefitting external behavioural problems in girls, rather than internal |
